# Effectiveness of Respiratory Muscles Training by Voluntary Isocapnic Hyperpnea Versus Inspiratory Threshold Loading on Intercostales and Vastus Lateralis Muscles Deoxygenation Induced by Exercise in Physically Active Adults

**DOI:** 10.3390/biology12020219

**Published:** 2023-01-30

**Authors:** Maximiliano Espinosa-Ramírez, Santiago Riquelme, Felipe Araya, Guido Rodríguez, Fernanda Figueroa-Martínez, Luigi Gabrielli, Ginés Viscor, W. Darlene Reid, Felipe Contreras-Briceño

**Affiliations:** 1 Laboratory of Exercise Physiology, Department of Health Science, Faculty of Medicine, Pontificia Universidad Católica de Chile, Av. Vicuña Mackenna #4860, Santiago 7820436, Chile; 2 Laboratory of Voice, Department of Health Science, Faculty of Medicine, Pontificia Universidad Católica de Chile, Av. Vicuña Mackenna #4860, Santiago 7820436, Chile; 3 Advanced Center for Chronic Diseases (ACCDiS), Division of Cardiovascular Diseases, Faculty of Medicine, Pontificia Universidad Católica de Chile, Marcoleta #367, Santiago 8380000, Chile; 4 Physiology Section, Department of Cell Biology, Physiology and Immunology, Faculty of Biology, Universitat de Barcelona, 08028 Barcelona, Spain; 5 Department of Physical Therapy and Interdepartmental Division of Critical Care Medicine, University of Toronto, Toronto, ON M5G 2C4, Canada; 6 KITE Research Institute, Toronto Rehabilitation Institute, University Health Network, Toronto, ON M5G 2A2, Canada; 7 Millennium Institute for Intelligent Healthcare Engineering, Av. Vicuña Mackenna #4860, Santiago 7820436, Chile

**Keywords:** respiratory muscles, spectroscopy, near-infrared, sport, athletic training, oxygen consumption

## Abstract

**Simple Summary:**

Respiratory muscle training (RMT) improves physical performance through increased efficiency of the muscles implicated in respiration, an aspect that preserves the blood flow, nutrients, and oxygen supply to the locomotor muscles. Whether this effect depends on the RMT method has yet to be discovered. The RMTs most employed in healthy subjects are based on inspiratory Threshold-load training (ITL), which trains mainly inspiratory muscles, and voluntary isocapnic hyperpnea (VIH), which trains both inspiratory and expiratory muscles. With the emergence of non-invasive technology that allows the assessment of the balance between the oxygen muscle supply and consumption, or muscle deoxygenation, as a reflection of changes in the blood flow at the microvascular tissue level target, it is possible to contrast the effect of ITL and VIH on the deoxygenation of locomotor muscles (*m. vastus lateralis*) and respiratory muscles (*m. intercostales*) simultaneously during exercise. This study showed that the deoxygenation of the intercostal muscles decreased after eight weeks of RMT independent of the type of training, with no effect on the vastus lateralis deoxygenation.

**Abstract:**

Respiratory muscle training (RMT) improves physical performance, although it is still debated whether this effect depends on the type of training. The purpose of this study was to compare the effects of two different types of RMT, i.e., voluntary isocapnic hyperpnea (VIH) and inspiratory threshold loading (ITL), on the deoxygenation of *intercostal* (ΔSmO_2_-*m. intercostales*) and *vastus lateralis* (ΔSmO_2_-*m. vastus lateralis*) muscles during exercise. Twenty-four participants performed eight weeks of RMT by: (i) VIH (3 days·week^−1^ for 12 min at 60% maximal voluntary ventilation) or (ii) ITL (5 sets·week^−1^ of 30 breaths·minute^−1^ at 60% maximal inspiratory pressure). Cardiopulmonary exercise testing (CPET) included ΔSmO_2_ (the change from baseline to end of test) of *intercostal* and *vastus lateralis* muscles. After RMT, both groups showed decreased ΔSmO_2_-*m. intercostales* (VIH = 12.8 ± 14.6%, *p* = 0.04 (effect size, ES = 0.59), and ITL = 8.4 ± 9.8%, *p* = 0.04 (ES = 0.48)), without a coincident change of ∆SmO_2-_*m. vastus lateralis*. ITL training induced higher $\dot{V}$O_2-peak_ absolute values than VIH (mean Δ post–pre, ITL = 229 ± 254 mL·min^−1^ [95% CI 67–391] vs. VIH, 39 ± 153 mL·min^−1^ [95% CI −58–136.0], *p* = 0.01). In conclusion, both RMT improved the balance between supply and oxygen consumption levels of *m. intercostales* during CPET, with ITL also inducing an increase of aerobic capacity.

## 1. Introduction

During incremental physical exercise, lung ventilation ($\dot{V}$E) can increase ten times the level required at rest in healthy subjects [1]. This demands recruitment of accessory respiratory muscles (RMs) [2] which in turn increases the oxygen-uptake associated with respiration ($\dot{V}$O_2_-RMs) from 2% to 10% at maximal exercise [3]. This phenomenon enhances the blood flow ($\dot{Q}$) and nutrients to RMs, which could limit $\dot{Q}$ to locomotor muscles (respiratory muscle metaboreflex), and consequently contribute to the development of peripheral locomotor muscle fatigue [4,5,6,7,8].

A strategy that can attenuate the respiratory muscle metaboreflex is respiratory muscle training (RMT) [9,10,11]. The physiological mechanisms for this attenuation are not entire clear. Concerning, Witt et al. (2007) reported that a RMT program reduces the activity chemosensitive afferent within the inspiratory muscles, achieving similar physical performance at the expense of a lower cardiovascular response [9]. In addition, McConnell et al. (2006), demonstrated that the RMT reduced the effect of prior fatigue of RMs contributing to the preservation of locomotor blood flow [12]. However, no study has contrasted the different methods of RMT based on several physiological training principles and hence have a differential effect on the respiratory muscle metaboreflex.

In healthy participants, the most studied RMT methods are: (1) voluntary isocapnic hypernea (VIH), which induces endurance training of the expiratory and inspiratory muscles, and (2) inspiratory threshold loading (ITL), which employs an overload primarily focused on the inspiratory muscles [13]. Differences in their effects on the RMs performance (strength and endurance) have been reported. VIH primarily increases RMs endurance [14], whereas ITL increases the endurance and mainly the strength of the RMs [15]. In addition, Walterspacher et al. (2018) suggest that these RMT methods produce the highest electromyographic activation of inspiratory muscles, although they showed differences in the activation pattern. ITL induces greater electromyographic (EMG) activation of the *m.diaphragm* than VIH, while VIH is associated with increased EMG activation of intercostal muscles [16]. The difference in the muscle groups trained, their effects on RM performance, and their different EMG activation patterns support the proposition that the effects on respiratory muscle metaboreflex may vary depending on the type of RMT.

Accordingly, we have proposed using non-invasive devices that can determine the oxygen saturation of muscles (SmO_2_) based on a model of the supply-to-extraction of oxygen at microvascular level [17,18,19] which can be determined by continuous near-infrared spectroscopy (NIRS, 630–850 nm) that measures the changes in oxygenated hemoglobin (O_2_Hb) and myoglobin (O_2_mHb) [20]. This method has been extensively studied in locomotor muscles (*m. vastus lateralis*) [21] and recently in respiratory muscles (*m.intercostales*) [18,22,23,24,25]. The decrease in SmO_2_ during exercise test (deoxygenation) can be calculated as ΔSmO_2_ = SmO_2_ at baseline −SmO_2_ at maximal intensity. The SmO_2_ will decrease by increased oxygen consumption by skeletal muscle, and/or restricted blood flow to the active muscles [19]. Thus, an attenuation in the respiratory muscle metaboreflex will be reflected in lower ΔSmO_2_-*m. intercostales* with a greater ΔSmO_2_-*m. vastus lateralis* during incremental effort. Therefore, it emerges as a valid and accessible alternative to contrast the local effects of different RMT methods on RM oxygen levels during incremental and maximal exercise.

Thus, the aim of this study was to contrast the effect of two RMT types, VIH versus ITL, on the deoxygenation of *intercostales* (ΔSmO_2_-*m. intercostales*) and *vastus lateralis* (ΔSmO_2_-*m. vastus lateralis*) muscles, during incremental physical exercise. We hypothesised that VIH training would reduce of ΔSmO_2_-*m. intercostales* and increase ΔSmO_2_-*m. vastus lateralis* more than ITL during incremental physical exercise. This was based on VIH versus ITL training having greater similarity to the hyperpnea induced during endurance exercise, its recruitment of both inspiratory and expiratory muscles, and its greater activation of intercostal muscles. 

## 2. Materials and Methods

### 2.1. Participants

Twenty-four healthy and physically active adults (≥150 min of moderate or ≥75 min of vigorous physical activity by week) were recruited in a non-probability convenience sampling through social network advertising. They were stratified to equal numbers of males and females per group and randomly allocated by a 1:1 sequence (generated by a researcher external to the authors of this study) to two training groups: (a) voluntary isocapnic hyperpnea (VIH), and (b) inspiratory threshold loading (ITL). Participants (19 to 44 years) with body mass index (BMI) < 25 kg·m^−2^ were included, without a history of respiratory, cardiovascular, metabolic, musculoskeletal, or neoplastic diseases or without any infectious or inflammatory process at least two weeks prior to the beginning of the study. All participants were informed (in verbal and written forms) of the study procedures and signed informed consent forms. This study followed the Declaration of Helsinki [26] and was approved by the Ethics Committee of the Pontificia Universidad Católica de Chile (Institutional Review Board, protocol number: 210525001, date of approval: 9 September 2021). The Table 1 shows the characteristics of participants. 

### 2.2. Protocol 

Participants were tested at the Laboratory of Exercise Physiology from Pontificia Universidad Católica de Chile for baseline and post-training. Procedures were performed under constant laboratory environmental conditions (temperature 20 ± 2 °C; relative humidity, 40 ± 2%) and within a similar time frame (from 9:00–14:00 h). Participants were asked to avoid physical activities for 24 h before the measurements and to avoid alcohol, caffeine, and other stimulants and food for at least 3 h prior to the evaluations. Before the evaluations, the participants completed the Spanish short version of the International Physical Activity Questionnaire (IPAQ). The physical activity level (PA) in the last 7 days was determined according to the METs·min^−1^·week^−1^ performed by the participants [27]. According to this questionnaire, we considered 3.3 METs for light PA (walking), 4 METs for moderate PA, and 8 METs for vigorous PA [27]. 

### 2.3. Baseline and Post-Training Evaluations

Anthropometric characteristics were measured (weight, height, and body mass index). Subsequently, spirometry (Microlab, model ML3500, CareFusion, San Diego, CA, USA) was performed according to the American Thoracic Society (ATS)–European Respiratory Society (ERS) guidelines [28], utilizing the reference values of Knudson [29]. Finally, maximal inspiratory pressures (MIP) were evaluated using a pneumometer (Micro MRC, CareFusion, Traunstein, Germany) according to the protocol proposed by the ATS and ERS [28], utilizing the reference values of Black and Hyatt [30]. Participants were seated position and wore a nose clip. The MIP measurement was performed at least 3 times until the values varied less than 10% [28]. Respiratory endurance was also measured by an incremental respiratory endurance test (IRE) using a POWERbreathe plus^®^ threshold loading devices (POWERbreathe International Ltd., Southham, UK) according to the modified protocol of Cahalin [31]. This consisted of a series of 90 s at a respiratory rate (RR) of 30 breaths·min^−1^, with an initial load of 10% of the MIP and increased by 10% until task failure or inability to maintain the RR. 

### 2.4. Cardiopulmonary Exercise Testing (CPET)

The exhaled gases, oxygen-uptake ($\dot{V}$O_2_), carbon-dioxide production ($\dot{V}$CO_2_), and ventilatory variables (lung ventilation ($\dot{V}$E), tidal volume (Vt), and respiratory rate (RR)) were evaluated using an ergospirometer (MasterScreen CPX, Jaeger, Germany) with breath-by-breath method during an incremental cycle-ergometer protocol using magnetically braked (ViaSprint 150P, Ergoline GmH, Traunstein, Germany). The ergospirometer was calibrated according to the manufacturer instructions, by means of calibration gas (5.00% carbon dioxide, 15.99% oxygen and remainder nitrogen). The protocol consisted of a 1-min rest, 5-min warm-up period at 40 watts, followed by an increase of 20 watts every 2 min until exhaustion, despite standardized verbal stimuli [19]. The participants were requested to maintain a cadence between 70 and 90 rpm during CPET. The $\dot{V}$O_2-peak_ was calculated as the highest value obtained during the last 30-s of CPET, despite increasing the exercise intensity (<150 mL·min^−1^ of exercise) [32]. A cool-down of 4-min of submaximal exercise was performed before allowing the participants to rest. At baseline and throughout test, the heart rate (HR) (RS300, Polar Electro, Kempele, Finland), oxygen pulse saturation (SpO_2_) (WristOx_2_ 3150, Nonin Medical, Minnesota, USA), and blood pressure (BP) (SunTech Tango M2 stress test monitor, SunTech Medical, Morrisville, NC, USA) were measured. In addition, dyspnoea, lower limb fatigue symptoms, and rating of perceived effort were assessed during the CPET using the modified Borg scale [33].

### 2.5. Muscle Oxygen Saturation (SmO_2_)

The SmO_2_ was evaluated by continuous-wave near-infrared spectroscopy (NIRS, 630 to 850 nm) using non-invasive devices (MOXY, Fortiori, Design LLC, Hutchinson, MN, USA). This device measures the absorbance of infrared light by oxygenated hemoglobin and myoglobin (oxy (Hb + Mb)) as well as deoxygenated hemoglobin and myoglobin (desoxi [Hb + Mb]), at a microvascular level [34,35]. From these values, SmO_2_ was calculated using PeriPedal^®^ (PeriPedal, Indianapolis, IN, USA) at a sampling frequency of 2 Hz [36], from the *m.intercostales* (SmO_2_-*m. intercostales*) and *m. vastus lateralis* (SmO_2_-*m.vastus lateralis*), according to a previous protocol [18]. In brief, for *m. intercostales*, a MOXY^®^ device was placed on the seventh intercostal space at the anterior axillary line in the right thoracic area. To determine the level of SmO_2_ in the locomotor muscles, a second MOXY^®^ device was placed over the *m. vastus lateralis*, 5 cm lateral to the midline of the thigh and landmarked midway between the upper edge of the patella and the greater trochanter of the right femur. The devices were fixed to the skin with double-sided sticky tape and hypoallergenic skin tape. 

### 2.6. Respiratory Muscle Training Protocol (RMT)

**Figure 1:**
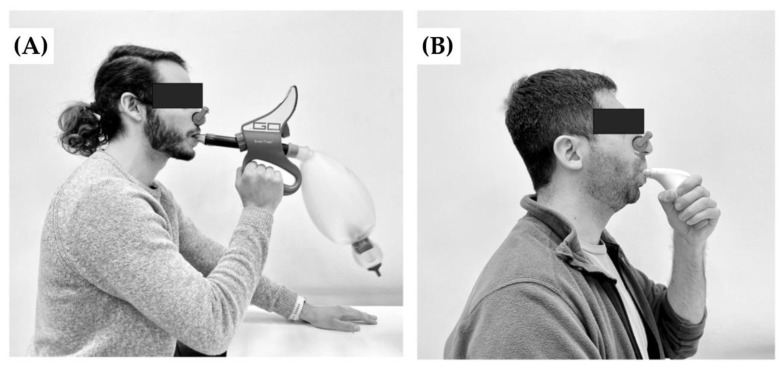
Type and devices used in the Respiratory Muscle Training (RMT): (**A**) Voluntary Isocapnic Hyperpnea (VIH) by SpiroTiger™; and (**B**) Inspiratory Threshold Loading (ITL) by POWERbreathe plus^®^.

RMT protocols were performed in the Laboratory of Exercise Physiology from Pontificia Universidad Católica de Chile and all training was supervised by the research team (see Figure 1). To compare HIV and ITL, we matched the weekly respiratory cycles of both training methods. For this purpose, we applied the formula: (respiratory rate × training time × number of weekly sessions × number of weeks of training). Thus, we considered 1080 cycles per week for each RMT protocol.

#### 2.6.1. Voluntary Isocapnic Hyperpnea Training (VIH)

VIH was carried out using the SpiroTiger^®^ device (SpiroTiger Sport, Indiag, Switzerland). This group completed 12-min training sessions, 3 sessions per week for 8 weeks with a load corresponding to 60% of the maximal voluntary ventilation (MVV) while maintaining a frequency of 30 breaths per minute according to the modified protocol of Leddy et al. [14]. 

#### 2.6.2. Inspiratory Threshold Loading Training (ITL)

ITL was carried out using the threshold loading with constant resistance with the POWERbreathe plus^®^ device (POWERbreathe International Ltd., Southam, UK) according to the modified protocol of Johnson et al. [37]. This group completed 30 dynamic inspiratory maneuvers per 7 min and 20 s (total time = 440 s) training sessions, during 5 sessions for 8 weeks, with a load corresponding to 60% of MIP. The load was modified weekly according to changes in the MIP. 

### 2.7. Data Analysis

Data for variables are presented as absolute values and as differences (Δ). To calculate peak oxygen consumption ($\dot{V}$O_2-peak_), the maximum value obtained during the last 30 s of the CPET was determined. For the absolute values, the average values obtained in the last 30 prior to $\dot{V}$O_2-peak_ values were calculated. For calculations of change (Δ), the difference was determined from average of the last 30 s of the resting stage (rest value) and the average of the last 30 s of maximum value. These differences were stated as positive or negative changes depending on whether there was an increase or decrease, respectively. In addition, related variables were calculated, where the primary outcome (ΔSmO_2_) was divided by different secondary outcome, e.g., change in lung ventilation (Δ$\dot{V}$E). 

### 2.8. Statistical Analysis

Normality of data was evaluated using the Shapiro–Wilk test. The descriptive variables are shown as mean ± standard deviation. The sample size was calculated from data extracted from pilot evaluations. Considering ∆SmO_2_-*m. intercostales* as the primary outcome, an effect size of 0.60, a power of 80% and 95% confidence, provided an estimated requirement of at least 24 subjects. The sample size calculation was performed using G*Power (version 3.1; Düsseldorf, Germany). Baseline values were compared by *t*-test for independent samples. The differences between the two methods of RMT, were analysed using the two-way mixed ANOVA test, reporting the interaction of the factors (group × time) and the effect of each of the factors: time (pre vs. post) and group (VIH vs. ITL). Subsequent multiple comparisons were analysed used the Bonferroni post-hoc test. Data are shown as mean difference ± standard deviation of mean differences. Moreover, effect sizes (ES) were calculated for the changes over time for each variable, by calculating Cohens d which were interpreted as 0 ≤ 0.2 *trivial*, 0.2 ≤ *small* < 0.5, 0.5 ≤ *moderate* < 0.8, 0.8 ≤ *large* ≤ 1.3, *very large* ≥ 1.3 [38]. In addition, ES of the time, group, and group × time effects were calculated using partial η^2^. ES obtained from η^2^ was considered *large* if ≥0.14, *moderate* ≥ 0.06, and *small* if <0.06 [38]. The correlations were assessed using Pearson’s correlation coefficient. Statistical significance was set at *p* < 0.05. The statistical analysis was performed using the GraphPad Prism (version 9.0; San Diego, CA, USA).

## 3. Results

Table 2 shows the data of the variables measured during CPET, with the values corresponding to the mean of the last 30 s of the maximal intensity of exercise.

### 3.1. Muscle Oxygen Saturation Levels

#### 3.1.1. Deoxygenation of *m. intercostales* (∆SmO_2_-*m. intercostales*, %)

**Figure 2:**
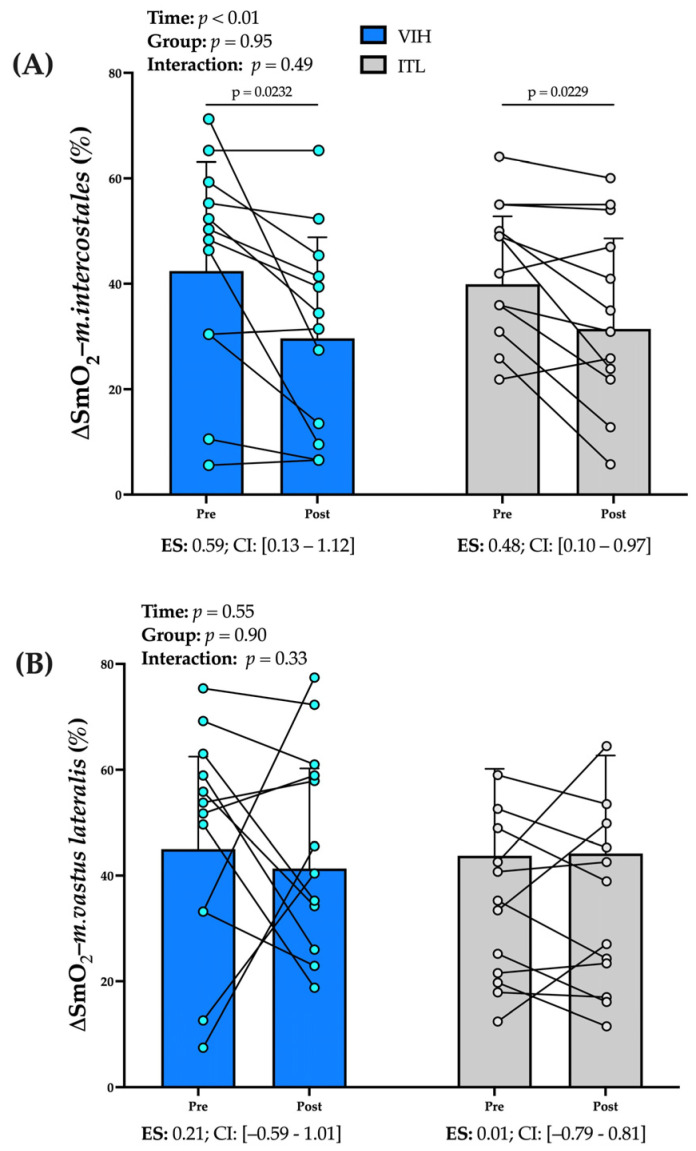
Comparison on changes in desoxygenation (ΔSmO_2_) of (**A**) *intercostales* muscles (ΔSmO_2-_*m.intercostales*, %), and (**B**) *vastus lateralis* muscles (ΔSmO_2-_*m. vastus lateralis*, %). Data were shown in mean ± SD. The factors (time and group) and their interaction are shown (group × time). Effect size (ES) are shown with confidence interval (CI) at 95%.

No significant interaction effect (group × time) was found for ∆SmO_2_-*m. intercostales* (*p* = 0.49), and only time factor (pre-post training) impacted it (*p* < 0.01). Eight weeks of training induced a smaller ∆SmO_2_-*m. intercostales* from pre to post-test for both RMT groups (VIH = 12.8 ± 14.6% [95% CI 3.5–22.0], *p* = 0.04 and ITL= 8.4 ± 9.8% [95% CI 2.2–14.7], *p* = 0.04); VIH reached a moderate effect size (ES = 0.59), whereas ITL induced a small effect size (ES = 0.48) (see Figure 2A).

#### 3.1.2. Deoxygenation of *m. vastus lateralis* (∆SmO_2-_m. *vastus lateralis*, %):

Neither method of RMT changed the ∆SmO_2-_*m. vastus lateralis* after the training (VIH, *p* = 0.56 vs. ITL, *p* = 0.99) (see Figure 2B).

### 3.2. Total hemoglobin 

Total hemoglobin for *m. intercostales* and *m. vastus lateralis* did not differ from pre-training to post-training for both RMT methods (see Table 2). 

### 3.3. SmO_2_ Ratio (∆SmO_2_-m. intercostales·∆SmO_2_-m. vastus lateralis^−1^)

There were no differences in SmO_2_ ratio from both RMT methods (VIH *p* = 0.25 vs. ITL *p* = 0.76) (see Table 2).

### 3.4. Cardiopulmonary Exercise Testing (CPET) 

**Figure 3:**
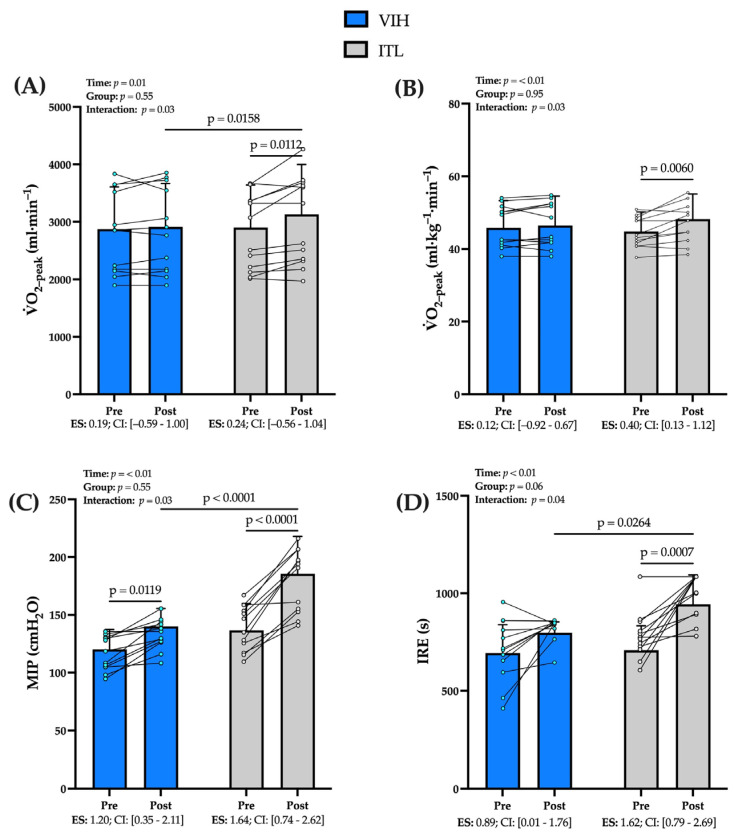
Comparison on changes in (**A**) absolute oxygen uptake ($\dot{V}$O_2-peak_, ml·min^−1^), (**B**) relative oxygen uptake ($\dot{V}$O_2-peak_, mL·kg^−1^·min^−1^), (**C**) *m.vastus lateralis* (ΔSmO_2-_*m. vastus lateralis*, %). Data are shown in mean ± SD. The factors (time and group) and their interaction are shown (group × time). Effect size (ES) are shown with confidence interval (CI) at 95%.

Compared to pre-training, ITL training induced higher $\dot{V}$O_2-peak_ absolute values than VIH (mean Δ post–pre, ITL = 229 ± 254 mL·min^−1^ [95% CI 67–391] vs. VIH, 39 ± 153 mL·min^−1^ [95% CI −58–136.0], *p* = 0.01) (see Figure 3A). Furthermore, only the ITL group increased $\dot{V}$O_2-peak_ relative (mean Δ post–pre, ITL = 3.4 ± 3.5 [95% CI 1.2–5.6] mL·kg^−1^·min^−1^, *p* < 0.01 vs. VIH = 0.7 ± 2.2 mL·kg^−1^·min^−1^ [95% CI −0.7–2.1], *p* = 0.84) compared to the values achieved in the pre-training CPET (see Figure 3B). These values translate to a small effect size (ES = 0.40) in $\dot{V}$O_2-peak_ for ITL training compared a trivial effect size (ES = 0.12) for VIH. Regarding the change in peak workload (watts) in response to training, there were no differences from both RMT methods (watts at peak stage and normalized to body weight (PtW)). Concerning ventilatory variables, only the ITL group improved their ventilatory efficiency ($\dot{V}$E·$\dot{V}$CO_2_^−1^) compared to the pre-training CPET (mean Δ pre–post, 2.1 ± 2.1 [0.7–3.4], *p* = 0.02), which represents a small effect size (ES = 0.30). Moreover, there were no differences in peak lung ventilation ($\dot{V}$E_-peak_) and peak respiratory rate (RR_-peak_) between groups. However, peak tidal volume (Vt_-peak_) decreased in ITL (mean Δ pre–post, 0.24 ± 0.22 L [95% CI −0.38–−0.09], *p* = 0.03), representing a small effect size (ES = 0.21) (see Table 2). 

### 3.5. Performance of Respiratory Muscle

ITL training achieved higher MIP than VIH training (mean Δ post–pre, ITL = 48 ± 23 cmH_2_O [95% CI 34–63] vs. VIH, 19 ± 13 cmH_2_O [95% CI 12–28], *p* = 0.01) (see Figure 3C). These values translate to a very large effect size (ES = 1.64) for ITL compared a large effect size (ES = 1.20) for VIH. Regarding the IRE, ITL training achieved higher values compared to VIH training (mean Δ post–pre, ITL = 216 ± 166 s [95% CI 111–322] vs. VIH, 104 ± 135 s [95% CI 18–190], *p* = 0.01) with a very large effect size (ES = 1.69) compared to the large effect size (ES = 0.89) for VIH (see Figure 3D). 

### 3.6. Deoxygenation Relative to Lung Ventilation and Peak Workload-to-Weight

The ∆SmO_2_-*m. intercostales*·∆$\dot{V}$E^−1^ decreased only in the VIH group (mean Δ pre–post 0.15 ± 0.16 [95% CI 0.04–0.25], *p* = 0.03). Both types of RMT did not affect ∆SmO_2_-*m. vastus lateralis*·PtW^−1^ (see Table 2).

### 3.7. Correlations

#### 3.7.1. Voluntary Isocapnic Hyperpnea Training (VIH)

**Figure 4:**
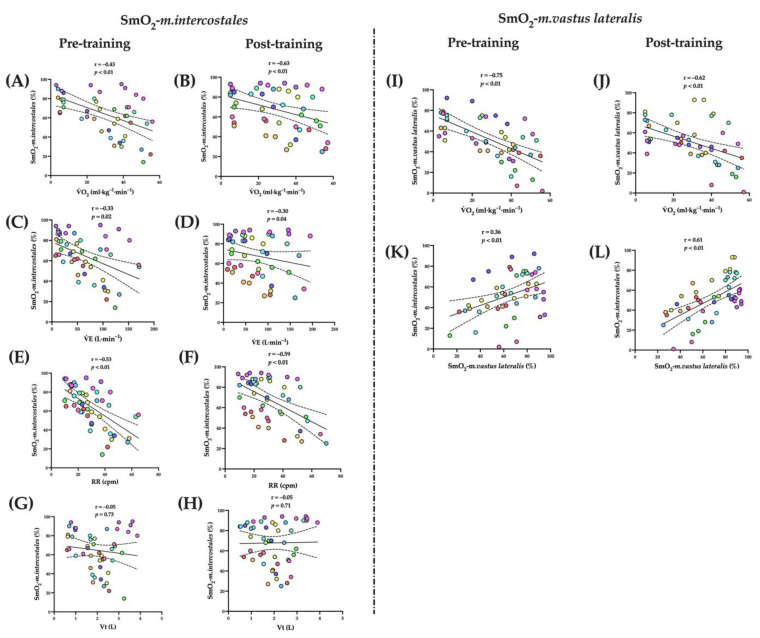
Correlations in VIH group of SmO_2_-*m. intercostales* (%) with $\dot{V}$O_2-peak_ (mL·kg^−1^·min^−1^) in pre- (**A**) and post-training (**B**); $\dot{V}$E (L·min^−1^) in pre- (**C**) and post-training (**D**); RR (cpm) in pre- (**E**) and post-training (**F**); and Vt (**L**) in pre- (**G**) and post-training (**H**). Correlation in VIH group of SmO_2_-*m.vastus lateralis* (%) with $\dot{V}$O_2-peak_ (mL·kg^−1^·min^−1^) in pre- (**I**) and post-training (**J**); and SmO_2_-*m. intercostales* (%) in pre- (**K**) and post-training (**L**). Abbreviations: SmO_2_ = muscle oxygen saturation; $\dot{V}$O_2-peak_ = peak oxygen uptake; $\dot{V}$E = lung ventilation; RR = respiratory rate; Vt = tidal volume.

The SmO_2-_*m.intercostales* (%) was inversely associated with $\dot{V}$O_2_ (mL·kg^−1^·min^−1^) in pre- (r = −0.43, *p* < 0.01) and post-training (r = −0.46, *p* < 0.01); $\dot{V}$E (L·min^−1^) in pre- (r = −0.33, *p* = 0.02) and post-training (r = −0.30, *p* = 0.04); RR (cpm) in pre- (r = −0.53, *p* < 0.01) and post-training (r = −0.59, *p* < 0.01). The SmO_2_-*m.vastus lateralis* was inversely associated with $\dot{V}$O_2_ (mL·kg^−1^·min^−1^) in pre- (r = −0.75, *p* < 0.01) and post-training (r = −0.62, *p* < 0.01), and was directly associated with SmO_2-_*m.intercostales* (%) in pre- (r = 0.61, *p* < 0.01) and post-training (r = 0.36, *p* < 0.01) (see Figure 4).

#### 3.7.2. Inspiratory Thresholds Loading Training (ITL)

**Figure 5:**
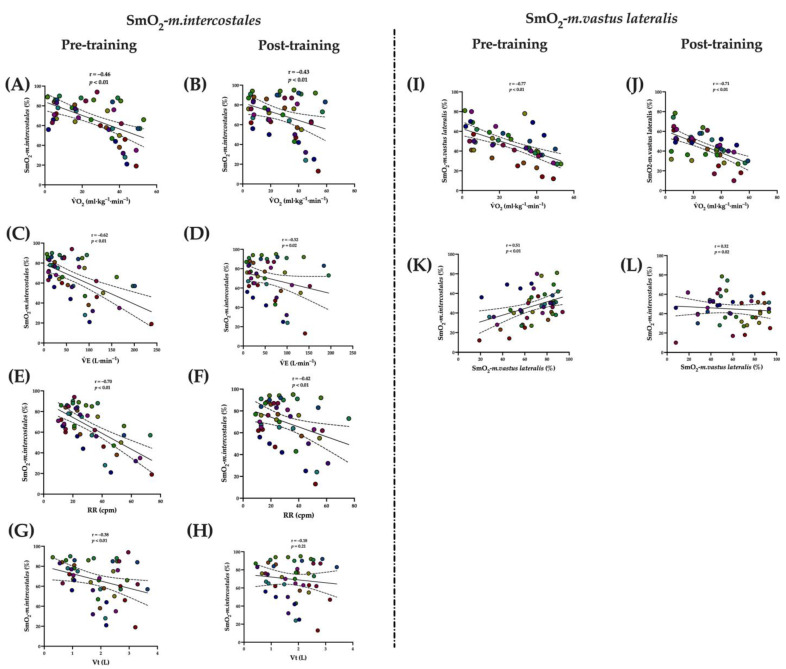
Correlations in ITL group of SmO_2_-*m. intercostales* (%) with $\dot{V}$O_2-peak_ (mL·kg^−1^·min^−1^) in pre- (**A**) and post-training (**B**); $\dot{V}$E (L·min^−1^) in pre- (**C**) and post-training (**D**); RR (bpm) in pre- (**E**) and post-training (**F**); and Vt (**L**) in pre- (**G**) and post-training (**H**). Correlation in VIH group of SmO_2_-*m.vastus lateralis* (%) with $\dot{V}$O_2-peak_ (mL·kg^−1^·min^−1^) in pre- (**I**) and post-training (**J**); and SmO_2_-*m.intercostales* (%) in pre- (**K**) and post-training (**L**). Abbreviations: SmO_2_ = muscle oxygen saturation; $\dot{V}$O_2-peak_ = peak oxygen uptake; $\dot{V}$E = lung ventilation; RR = respiratory rate; Vt = tidal volume.

The SmO_2_-*m. intercostales* (%) was inversely associated with $\dot{V}$O_2_ (mL·kg^−1^·min^−1^) in pre- (*r* = −0.46, *p* < 0.01) and post-training (*r* = −0.43, *p* < 0.01); $\dot{V}$E (L·min^−1^) in pre- (*r* = −0.62, *p* < 0.01) and post-training (*r* = −0.32, *p* = 0.02); RR (cpm) in pre- (*r* = −0.70, *p* < 0.01) and post-training (*r* = −0.42, *p* < 0.01); and Vt only in pre-training (*r* = −0.38, *p* < 0.01). The SmO_2-_*m. vastus lateralis* was inversely associated with $\dot{V}$O_2_ (mL·kg^−1^·min^−1^) in pre- (*r* = −0.77, *p* < 0.01) and post-training (*r* = −0.71, *p* < 0.01), and was directly associated with SmO_2-_*m. intercostales* (%) in pre- (*r* = 0.51, *p* < 0.01) and post-training (*r* = 0.32, *p* = 0.02) (see Figure 5).

## 4. Discussion

The main objective of our study was to contrast the effect of two different RMT, VIH and ITL, on the deoxygenation of *m. intercostales* (∆SmO_2_-*m. intercostales* = SmO_2_-*m. intercostales*_rest_–SmO_2_-*m. intercostales*_max_) and *m. vastus lateralis* (∆SmO_2_-*m. vastus lateralis* = SmO_2_-*m. vastus lateralis*_rest_–SmO_2_-*m. vastus lateralis*_max_) during maximal incremental exercise. In contrast to our hypotheses, after eight weeks of training, both groups decreased the ∆SmO_2_-*m. intercostales* without any effect on the ∆SmO_2_-*m. vastus lateralis*. Furthermore, ITL group demonstrated greater physical conditioning compared to the VIH group as reflected by a significant increase in $\dot{V}$O_2-peak_ (relative and absolute), as well as more significant RM-specific improvements in their strength, endurance, and efficiency.

### 4.1. Effect of RMT in ∆SmO_2_-m. intercostales and ∆SmO_2_-m. Vastus Lateralis

Previous studies have reported that during high intensity exercise there is a competition of blood flow between respiratory and locomotor muscles (Respiratory muscle metaboreflex) [39,40]. While NIRS devices do not directly assess blood flow, they provide a measurement of the balance between oxygen supply and consumption at the muscular level [20,41]. So, we postulated that RMT, by attenuating the respiratory muscle metaboreflex would be reflected in a low ∆SmO_2_-*m. intercostales* and high ∆SmO_2_-*m. vastus lateralis* during incremental exercise. In addition, because there are different methods of RMT, we propose that VIH training (inspiratory–expiratory) would have a greater effect on SmO_2_. However, only significant changes were found in the ∆SmO_2_-*m. intercostales*, and this effect was independent of the type of training (VIH = 12.8% and ITL: 8.4%). Our findings, shows that eight weeks of RMT may decrease this muscle’s oxygen requirement and/or improve its matching of oxygen delivery to the demands of oxygen consumption of *m. intercostales*. This comparison is novel evidence given that previous studies only have reported positive effects of ITL on $\dot{V}$O_2-RMs_. In this regard, Turner et al. (2012) demonstrated that a six-week ITL program reduced the cost of breathing during voluntary hyperpnoea in physically active subjects, decreasing $\dot{V}$O_2-RM_ by 3.4%. They postulated that ITL can reduce the energy requirements of the RM_S_ while maintaining high levels of $\dot{V}$E [11]. Likewise, other authors have reported that ITL improves the dynamics of $\dot{V}$O_2-RMs_, which decrease the fatigue and metabolic requirements of the RMs while maintaining high levels of $\dot{V}$O_2-peak_ [42]. Accordingly, ITL training reduces the oxygen requirements and optimizes the performance of the RMs likely by augmenting aerobic metabolism [43]. In addition, previous studies have reported that a decrease in RMs oxygen requirements implies an improvement in exercise tolerance, which is associated with an attenuation of the metaboreflex [9,10], i.e., increases blood flow available for locomotor musculature (LM_S_). However, neither of our RMT programs induced effects on ∆SmO_2_-*m. vastus lateralis*, which suggests the need to include training protocols involving lower limb activation in conjunction with RMT in future research.

### 4.2. Effect of RMT in Physical Performance

Regarding systemic effects, only the ITL group showed a significant increase in $\dot{V}$O_2-peak_, reaching in average 191 mL·min^−1^ more than the VIH group. Moreover, only ITL training increased $\dot{V}$O_2-peak_ in absolute (228 mL·min^−1^) and relative (3.4 mL·kg^−1^·min^−1^) values (Figure 3), which represented small effect sizes (absolute ES = 0.24 and relative ES = 0.40). These results contrast with previous studies, which have reported no increase in $\dot{V}$O_2_ following RMT [14,15]. These differences may be due to the volume, planning, and supervision of both training protocols. In addition, our findings are of great relevance since, as mentioned above, ITL decreased the ΔSmO_2_-*m. intercostales* which was associated with an increase in maximal aerobic capacity (ITL, *r* = −0.63, *p* < 0.01), and thus a better physical performance of a maximal exercise test. This association could be explained by ITL inducing a greater activation of diaphragm, which remains in constant contraction at high physical effort, a desirable attribute for those striving to improve physical performance [44]. Further, other studies have reported that both RMT programs improve $\dot{V}$O_2-peak_ independent of the type of training, especially in participants with lower physical capacity [45]. However, it should be noted that there is little evidence comparing the effect of both types of training with similar inspiratory loads on $\dot{V}$O_2-peak_. Regarding inspiratory load, ITL training has been shown to be more effective than VIH training in disciplines with higher inspiratory muscle stress, such as swimming, improving competition times and sporting performance, so differing findings may be explained by the unique test characteristics (incremental and maximal) that lead to high stress to the respiratory system in a short period [46].

### 4.3. Effect of RMT in Respiratory Muscle Performance

In addition to the systemic effects, the ITL group showed greater RM-specific effects reflected by higher strength, endurance and ventilatory efficiency values. In this regard, our results reflect an increase of 48 cmH_2_O in peak inspiratory pressure (MIP) in the ITL group, an increase of 45 cmH_2_O more than that achieved by the VIH group. Notably, both groups achieved large effect sizes (ITL, ES = 1.64 and VIH, ES = 1.20). These findings are consistent with previous work by Van Hollebeke et al. (2020), who reported increase in MIP after ITL and tapered flow resistive loading after six weeks of training in healthy subjects [47]. Regarding respiratory muscle endurance, our results agree with previous reports in the literature, as it improved in both VIH and ITL training.

We found that higher MIP values do not necessarily translate to improved physical performance. Our findings contrast a previous study that found VIH training to induce greater increases in running duration until exhaustion, although these studies were evaluated in a sample of high-performance athletes, with fewer participants (n = 8), and after a shorter training program (four weeks) [46]. In contrast, our ITL group increased respiratory muscle endurance by 217 s compared to baseline values, representing a very large effect size (ES = 1.69), while no significant differences were reported in the VIH group. This type of RMT seeks to maintain high $\dot{V}$E levels to replicate the RM effort necessary to sustain intensities like aerobic endurance training. However, previous studies reported that ITL generates greater electromyographic activation of the diaphragm and chest wall muscles compared to other RMT strategies [48], which could translate to training a larger motor neuronal pool with ITL compared to VIH. These differences in respiratory performance were only associated in the ITL group, as shown by increased ventilatory efficiency (mean difference pre–post, 2.1 the $\dot{V}$E·$\dot{V}$CO_2-nadir_^−1^), a variable reflective of improved CO_2_ removal, although its implications for improved fitness after training are not yet defined [49].

### 4.4. Limitations 

The main limitation of this study was the non-inclusion of a placebo group. This comparison would have allowed us to attribute the effects to the training, discarding the influence of time and attention. We therefore suggest including a sham intervention group as a third comparison in future studies. Our study evaluated the changes in oxygen saturation in *intercostales* and *vastus lateralis* muscles during incremental exercise by using the MOXY^®^ device. This equipment does not differentiate the changes in oxygenated and deoxygenated haemoglobin, variables that represent the extraction of oxygen at a muscular level [50]. Future studies incorporating NIRS devices that allow evaluation of these physiological variables could expand the results obtained. Likewise, another variable to consider is the penetration of the light signal to the target muscle, which is deemed half the distance between the optodes (emitter and receiver) and was 1.5 cm for the MOXY^®^ device [51]. Ultrasound evaluation of adipose tissue thickness could further verify NIRS evaluation of the target muscle. Regardless, our sample participants all had normal BMI (VIH: 22 ± 1 kg·m^−2^ and ITL: 21 ± 1 kg·m^−2^), which reduces the probability of error in the recording from our device. Other limitations of this study are possible confounding variables. Since it was not possible to perform a direct assessment of the work of breathing (WOB) by esophageal catheterization in our experimental set-up, the possibility that RMT causes an increased work of the respiratory muscles, although unlikely under the conditions of our study, cannot be completely rules out. For this reason, in future studies, we suggest that include the direct assessment of the WOB. Moreover, although all training sessions were performed with supervision by at least one member of the research team, potential changes in nutritional habits and physical activity during the training period were not controlled. For example, exceeding 300 min of physical activity per week could induce changes in physical performance that are independent of the RMT programs. Similarly, it is suggested in future projects to objectively record the level of previous physical activity of the participants, a variable that could affect the initial performance of the participants. However, it should be noted that our study did not show significant differences in pre-training $\dot{V}$O_2-peak_ for either of the two training groups. We therefore suggest for future studies to objectively assess the level of physical activity by accelerometry. In addition, another limitation of the study was the number of VIH training sessions. Previous studies have reported that favourable results are found with five days a week training. Therefore, we suggest modifying this protocol in future studies by increasing the number of sessions per week [52]. 

Finally, since neither of the two types of training showed effects on ∆SmO_2_-*m. vastus lateralis*, in addition to the fact that combined training programs have shown better results on exercise tolerance in various populations, we suggest evaluating the impact of an integrated limb muscle and RM training program on respiratory and locomotor muscle oxygenation. It would also be interesting to study the effect of these RMT programs on muscle oxygen levels in other populations, such as athletes, participants with chronic diseases, and children, in whom the evidence is scarce and could provide a new variable to support the phenomena of sports performance, survival, and development.

## 5. Conclusions

An eight-week RMT decreases the intercostal muscle deoxygenation regardless of the type of training used, but the ITL induced better physical conditioning and performance of respiratory muscles. However, neither type of training affected the deoxygenation of locomotor muscles. In future studies, we suggest the implementation of training programs that combine RMT and lower limb training by extending the sample to participants with diverse conditions, such as athletes, patients, and sedentary subjects.

## Figures and Tables

**Table 1:** Baseline characteristics.

	VIH (n = 12)	ITL (n = 12)	*p*-Value	[95% CI]
	Mean ± SD	Mean ± SD		
Sex (men/women)	6/6	6/6	-	-
Age (years)	22 ± 1	21 ± 1	0.45	[−1.2–0.6]
Height (cm)	169 ± 7	169 ± 11	0.90	[−7.6–6.7]
Weight (kg)	62 ± 9	64 ± 12	0.63	[−6.3–10.2]
BMI (kg⋅m^−2^)	21.6 ± 1.6	22.3 ± 2.6	0.33	[−0.8–2.1]
Physical activity (min·week^−1^)				
Light	81 ± 21	83 ± 27	0.86	[−18.6–22.1]
Moderate	223 ± 29	236 ± 24	0.23	[−9.4–36.1]
Vigorous	83 ± 25	82 ± 26	0.90	[−22.6–20.1]
Total	387 ± 44	401 ± 33	0.39	[−19.3–46.9]
FVC (L)	4.9 ± 0.7	4.8 ± 1.8	0.74	[−0.8–0.6]
FEV_1_ (L)	4.1 ± 0.5	4.0 ± 1.5	0.78	[−0.6–0.5]
FEV_1_⋅FVC^−1^ (%)	82.6 ± 4.6	83.3 ± 5.2	0.71	[−3.1–4.5]
MIP (cm H_2_O)	120 ± 17	138 ± 24	0.06	[−0.7–33.8]
IRE (s)	694 ± 146	707 ± 127	0.81	[−102–129]
CPET pre-RMT				
$\dot{V}$O_2-peak_ (mL·kg^−1^·min^−1^)	46 ± 8	45 ± 6	0.71	[−6.5–4.5]
Heart rate-_peak_ (bpm)	182 ± 8	181 ± 9	0.94	[−7.3–6.8]
Workload_-peak_ (watts)	215 ± 46	218 ± 58	0.90	[−41–46]
Time to exhaustion (s)	822 ± 189	843 ± 223	0.80	[−153–196]
Respiratory rate_-peak_ (cpm)	48 ± 10	55 ± 13	0.15	[−2.6–15.9]
Tidal volume_-peak_ (L)	2.6 ± 0.6	2.6 ± 1.4	0.84	[−0.6–0.5]
Lung ventilation_-peak_ (L·min^−1^)	123 ± 28	142 ± 51	0.29	[−16–52]
∆SmO_2-_*m. intercostales* (%)	42 ± 21	40 ± 14	0.73	[−17–12]
∆tHb-*m. intercostales* (g·dL^−1^)	0.2 ± 0.2	0.2 ± 0.5	0.80	[−0.4–0.3]
∆SmO_2-_*m. vastus lateralis* (%)	45 ± 18	44 ± 17	0.85	[−16–13]
∆tHb-*m. vastus lateralis* (g·dL^−1^)	0.2 ± 0.2	0.2 ± 0.3	0.64	[−0.3–0.2]

Data are presented as means ± standard deviations, with 95% confidence intervals. Abbreviations: VIH = voluntary isocapnic hyperpnea; ITL = inspiratory threshold loading; CPET = cardiopulmonary exercise testing; RMT = respiratory muscle training; BMI = body mass index; FVC = forced vital capacity; FEV_1_ = forced expiratory volume in the first second; MIP = maximal inspiratory pressure; IRE = incremental respiratory endurance; $\dot{V}$O_2-peak_ = peak oxygen-uptake; ∆SmO_2_ = muscle deoxygenation (SmO_2-rest–_SmO_2-peak_); ∆tHb = difference of total hemoglobin (tHb_-rest_ − tHb_-peak_).

**Table 2:** Changes in CPET, Spirometry, respiratory muscle performance, muscle oxygenation, and related variables.

Variable	Training Groups						Two-Way ANOVA Results (*p*-Values)							
	VIH			ITL			Factor				Interaction		Multiple Comparison	
	Pre (Mean ± SD)	Post (Mean ± SD)	Effect Size	Pre (Mean ± SD)	Post (Mean ± SD)	Effect Size	Time	Effect Size	Groups	Effect Size	Time × Group	Effect Size	VIH_-pre_ vs. VIH_-post_ / ITL_-pre_ vs. ITL_-post_	VIH_-pre_ vs. ILT_-pre_ / VIH_-post_ vs. ILT_-post_
***CPET test***														
$\dot{V}$O_2-peak_ (mL·kg^−1^·min^−1^)	46 ± 8	47 ± 8	0.12	45 ± 6	48 ± 8	0.40	<0.01 **	0.56	0.95	0.04	0.02 *	0.37	0.82/<0.01 **	0.58/0.16
$\dot{V}$O_2-peak_(mL·min^−1^)	2874 ± 732	2721 ± 754	0.19	2902 ± 739	3104 ± 897	0.24	0.01 *	0.51	0.55	0.46	0.03*	0.34	0.89/0.01 *	0.95/0.01 *
Peak workload (watts)	215 ± 46	224 ± 49	0.18	216 ± 58	220 ± 57	0.07	0.09	0.26	0.96	0.00	0.29	0.10	-	-
Peak workload-to-weight	3.4 ± 0.5	3.6 ± 0.5	0.38	3.4 ± 1.2	3.4 ± 1.2	0.03	0.06	0.29	0.53	0.47	0.30	0.08	-	-
Time to exhaustion (s)	822 ± 189	858 ± 198	0.17	844 ± 223	853 ± 222	0.04	0.09	0.57	0.90	0.14	0.05	0.30	-	-
Heart rate_-peak_ (bpm)	182 ± 8	188 ± 7	0.77	181 ± 9	184 ± 11	0.29	0.09	0.23	0.46	0.07	0.43	0.06	-	-
∆Heart rate (bpm)	96 ± 8	106 ± 13	0.89	99 ± 19	107 ± 14	0.46	<0.01 **	0.65	0.67	0.06	0.68	0.02	0.01 */0.04 *	-
Respiratory rate_-peak_ (cpm)	48 ± 10	53 ± 9	0.37	55 ± 13	53 ± 11	0.13	0.53	0.03	0.36	0.23	0.17	0.16	-	-
∆Respiratory rate (cpm)	33 ± 9	38 ± 11	0.48	39 ± 13	38 ± 11	0.08	0.46	0.06	0.42	0.18	0.25	0.12	-	-
Tidal volume_-peak_ (L)	2.6 ± 0.6	2.5 ± 0.7	0.14	2.6 ± 1.4	2.3 ± 1.2	0.21	<0.01 **	0.44	0.67	0.24	0.34	0.08	0.39/0.03 *	-
∆Tidal volume (L)	1.7 ± 0.7	1.5 ± 0.6	0.29	1.8 ± 1.4	1.4 ± 1.2	0.29	0.02 *	0.32	0.94	0.00	0.40	0.06	0.76/0.18	-
Lung ventilation_-peak_ (L·min^−1^)	124 ± 28	130 ± 37	0.17	142 ± 51	125 ± 39	0.36	0.37	0.08	0.61	0.12	0.05	0.29	-	-
∆Lung ventilation (L·min^−1^)	110 ± 30	114 ± 36	0.12	130 ± 50	112 ± 38	0.39	0.31	0.11	0.47	0.19	0.07	0.27	-	-
Ventilatory efficiency	26 ± 3	26 ± 3	0.16	26 ± 5	25 ± 4	0.30	0.02 *	0.41	0.97	0.00	0.01 *	0.41	0.99/0.03 *	0.76/0.84
***Spirometry test***														
FEV_1_ (L)	4.1 ± 0.5	4.2 ± 0.5	0.26	4.0 ± 1.5	3.9 ± 1.5	0.06	0.08	0.26	0.42	0.47	0.92	0.00	-	-
FVC (L)	4.9 ± 0.7	4.9 ± 0.8	0.03	4.8 ± 1.8	4.6 ± 1.8	0.12	0.03 *	0.25	0.90	0.00	0.93	0.00	0.37/0.45	-
FEV_1_⋅ FVC^−1^ (%)	83 ± 5	86 ± 11	0.41	83 ± 5.1	85 ± 6	0.34	0.02 *	0.35	0.88	0.01	0.41	0.06	0.08/0.57	-
***Respiratory muscle performance***														
MIP (cmH_2_O)	120 ± 17	140 ± 15	1.20	137 ± 24	186 ± 33	1.64	<0.01 **	0.90	<0.01 **	0.88	<0.01 **	0.61	<0.01 **/< 0.01 **	0.02 */<0.01 **
MIP regard to predicted (%)	105 ± 14	124 ± 19	1.09	123 ± 20	166 ± 20	2.07	<0.01 **	0.90	<0.01 ***	0.90	<0.01 **	0.60	<0.01 **/<0.01 ***	<0.01 **/<0.01 **
IRE (S)	694 ± 146	797 ± 56	0.89	707 ± 127	924 ± 120	1.69	<0.01 **	0.76	0.06	0.41	0.04 *	0.32	0.16/<0.01 ***	0.98/0.01 *
***Muscle oxygenation***														
SmO_2_-*m. intercostales*_-peak_ (%)	43 ± 23	52 ± 26	0.35	43 ± 17	52 ± 26	0.39	<0.01 **	0.46	0.86	0.03	0.95	0.00	0.15/0.13	-
∆SmO_2_-*m. intercostales* (%)	42 ± 21	30 ± 19	0.59	40 ± 14	32 ± 18	0.48	<0.01 **	0.53	0.95	0.00	0.49	0.04	0.02 */0.02 *	-
SmO_2_-*m. vastus lateralis*_-peak_ (%)	37 ± 19	39 ± 21	0.09	34 ± 12	32 ± 11	0.17	0.52	0.02	0.88	0.00	0.18	0.02	-	-
∆SmO_2_-*m. vastus lateralis* (%)	45 ± 18	41 ± 19	0.21	44 ± 17	44 ± 19	0.01	0.55	0.06	0.91	0.01	0.31	0.09	-	-
tHb-*m. intercostales*_-peak_ (g·dL^−1^)	12.0 ± 0.3	12.1 ± 0.4	0.27	12.1 ± 1.3	12.2 ± 1.3	0.07	0.65	0.01	0.52	0.18	0.47	0.01	-	-
∆tHb-*m. intercostales* (g·dL^−1^)	0.2 ± 0.2	0.1 ± 0.2	0.48	0.2 ± 1.3	0.2 ± 1.0	0.01	0.55	0.03	0.42	0.00	0.98	0.02	-	-
tHb-*m. vastus lateralis*_-peak_ (g·dL^−1^)	12.1 ± 0.6	11.9 ± 0.6	0.32	12.0 ± 1.2	12.1 ± 1.1	0.08	0.28	0.15	0.71	0.13	0.02 *	0.42	0.07/0.77	0.74/0.08
∆tHb-*m. vastus lateralis* (g·dL^−1^)	0.2 ± 0.2	0.3 ± 0.2	0.48	0.2 ± 1.2	0.3 ± 0.9	0.09	0.42	0.10	0.77	0.05	0.52	0.04	-	-
***Related variables***														
∆SmO_2_-*m. intercostales* · ∆VE^−1^	0.4 ± 0.2	0.3 ± 0.2	0.48	0.4 ± 0.9	0.3 ± 1.0	0.10	<0.01 **	0.39	0.75	0.07	0.07	0.26	0.01 */0.99	-
∆SmO_2_-*m. vastus lateralis* · PtW^−1^	13 ± 4	11 ± 4	0.48	13 ± 5	13 ± 6	0.01	0.26	0.20	0.64	0.16	0.25	0.12	-	-
Ratio SmO_2_	1.1 ± 0.7	0.8 ± 0.6	0.48	1.1 ± 1.4	0.9 ± 1.5	0.13	0.02 *	0.28	0.79	0.03	0.49	0.05	0.14/0.70	-

Data are presented as means ± standard deviations, with 95% confidence intervals. *: *p* < 0.05 and **: *p* < 0.01 (statistical differences between groups, according to the two-way ANOVA test). Abbreviations: VIH = voluntary isocapnic hyperpnea; ITL = inspiratory threshold loading training; FVC = forced vital capacity; FEV_1_ = forced expiratory volume in the first second; MIP = maximal inspiratory pressure; IRE = incremental respiratory endurance; $\dot{V}$O_2-peak_, = peak oxygen-uptake; ∆SmO_2_ = muscle deoxygenation (SmO_2-rest_–SmO_2-peak_); ∆tHb = difference of total hemoglobin (tHb_-rest_–tHb_-peak_); PtW = Peak workload-to-weight; ratio SmO_2_ = ∆SmO_2_-*m. intercostales*_·_ SmO_2_-*m. vastus lateralis*^−1^.

## Data Availability

Not applicable.
